# Cluster of *Cryptococcus neoformans* Infections in Intensive Care Unit, Arkansas, USA, 2013

**DOI:** 10.3201/eid2110.150249

**Published:** 2015-10

**Authors:** Snigdha Vallabhaneni, Dirk Haselow, Spencer Lloyd, Shawn Lockhart, Heather Moulton-Meissner, Laura Lester, Gary Wheeler, Linda Gladden, Kelley Garner, Gordana Derado, Benjamin Park, Julie R. Harris

**Affiliations:** Author affiliations: Centers for Disease Control and Prevention, Atlanta, Georgia, USA (S. Vallabhaneni, S. Lloyd, S. Lockhart, H. Moulton-Meissner, L. Lester, G. Derado, B. Park, J.R. Harris); Arkansas Department of Health, Little Rock, Arkansas, USA (D. Haselow, L. Lester, G. Wheeler, L. Gladden, K. Garner)

**Keywords:** Cryptococcus, cluster, outbreak, ICU, steroids, fungi, intensive care, Cryptococcus neoformans, United States, Arkansas

## Abstract

We investigated an unusual cluster of 6 patients with *Cryptococcus neoformans* infection at a community hospital in Arkansas during April–December 2013, to determine source of infection. Four patients had bloodstream infection and 2 had respiratory infection; 3 infections occurred within a 10-day period. Five patients had been admitted to the intensive care unit (ICU) with diagnoses other than cryptococcosis; none had HIV infection, and 1 patient had a history of organ transplantation. We then conducted a retrospective cohort study of all patients admitted to the ICU during April–December 2013 to determine risk factors for cryptococcosis. Four patients with *C. neoformans* infection had received a short course of steroids; this short-term use was associated with increased risk for cryptococcosis (rate ratio 19.1; 95% CI 2.1–170.0; p<0.01). Although long-term use of steroids is a known risk factor for cryptococcosis, the relationship between short-term steroid use and disease warrants further study

## Abstract 2

Use of short-term steroids was a risk factor for infection.

*Cryptococcus neoformans* is an encapsulated yeast found in soil throughout the world, particularly in soil contaminated with pigeon guano (*1*). Persons become infected by inhaling fungal spores (*2*), and the infection is usually asymptomatic. In some persons, latent infections can be established in the lungs and hilar lymph nodes (*3*). Cryptococcal disease typically manifests when latent infection is reactivated after a person becomes immunosuppressed (e.g., receives long-term steroids or immunosuppressive medications for an organ transplant or has advanced HIV infection) (*4*). Meningitis and pneumonia are the most common manifestations, and bloodstream infection (BSI) occurs far less frequently (*5*). Because cryptococcosis rarely results from acute fungal exposure and because person-to-person transmission is exceedingly uncommon (*6*,*7*), clusters of patients with this disease are unusual.

Hospital A is a community hospital in northwestern Arkansas, USA, with ≈300 beds, 38 of which are intensive care unit (ICU) beds. *C. neoformans* was isolated from 6 patients during 2013: 4 patients had *C. neoformans* BSI, and 2 had bronchoalveolar lavage (BAL) specimens yielding *C. neoformans.* Three of the 4 *C. neoformans* BSI cases occurred within a 10-day period in April–May 2013. Although *C. neoformans* infection is not a reportable condition in Arkansas, an astute infection control practitioner (ICP) at hospital A noted the unusual number of *C. neoformans* BSIs in a short period and contacted the Arkansas Department of Health. Preliminary investigation revealed that most case-patients had been admitted to the hospital A ICU, which prompted concern that the ICU might be the source of acute cryptococcal infection. All case-patients died, most within days of their positive *C. neoformans* culture. This unusual set of circumstances—clustering in time of an uncommon disease among patients with exposure to a single hospital unit—combined with the high death rate led to an on-site investigation to further characterize clinical illness among patients in this cluster and identify possible sources of and risk factors for infection.

## Methods

A case was defined as culture-confirmed *C. neoformans* infection in an inpatient admitted to hospital A during 2013. We reviewed microbiology records at hospital A for 1992–2013 to identify all cases and establish a background rate for positive cryptococcal cultures at hospital A. We telephoned microbiology staff and ICPs at surrounding hospitals to inquire about any recent changes noted in rates of isolation of *Cryptococcus* spp. 

Case-patients’ medical records were reviewed, and data were abstracted by using a standardized case report form that included demographic and clinical information. Case-patients’ family members (or case-patients, if alive) were interviewed by using a standardized questionnaire to identify potential exposures to *Cryptococcus. *Questions included whether patient had contact with pigeons or other birds and whether case-patients had engaged in any common activities in the community that could have resulted in a common acute exposure to *C. neoformans* before hospital admission. We interviewed laboratory managers and technicians at hospital A to assess specimen collection and processing methods and to identify any potential sources for contamination of specimens with *C. neoformans*.

To assess the possibility of acute, nosocomially acquired *C. neoformans* infection, we took several steps. Case-patient charts were reviewed for commonalities in physical location, procedures, and providers. ICU staff, hemodialysis staff, and ICPs at hospital A were interviewed to gain an understanding of how patients are cared for in the ICU, dialysis procedures, and infection control practices. We also asked hospital leadership about any known history of illegal activity or recent disciplinary action of ICU staff members. We asked facilities management staff about any bird habitats at the hospital and recent construction activity. Because *C. neoformans* thrives in bird guano, we also interviewed all ICU staff members about contact with birds and sampled the hands using Handi-wipes (*8*) and homes using Sponge-Sticks (3M Co., St. Paul, MN, USA) and vacuum filter socks (X-Cell 100 Dust Sampling Sock, Midwest Filtration Co, Cincinnati, OH, USA) (*9*) of ICU staff members who reported substantial bird exposure and had contact with case-patients. Environmental sampling was conducted in the ICU with Sponge-Stick swabs.

Finally, we conducted a retrospective cohort study to identify factors associated with cryptococcosis. Patients admitted to the hospital A ICU during April 1–December 31, 2013 (the period during which the cases occurred), were included. Patients were identified by querying the electronic medical record database. Data extracted from the medical record included length of stay in the ICU, receipt of glucocorticoids in the ICU, type of steroid if one was administered, and whether the patient was cared for by a specific respiratory therapist. For the purposes of the cohort analysis, only case-patients who were admitted to the ICU were included (n = 5). Poisson regression models were fit to the data to examine the relationship between the potential risk factors and cryptococcosis and to estimate the rate ratios between different exposure groups.

Clinical isolates were confirmed as *C. neoformans* by melanin production on L-DOPA media and by lack of growth after inoculation on canavanine-glycine-bromothymol blue media (*10*). Isolates were subtyped by using multilocus sequence typing (MLST). The *URA5, IGS1, CAP59, LAC1, GPD1, PLB1,* and *SOD1* gene fragments were amplified as described (*11*) for all isolates. Allele numbers and sequence types were determined by using the online *C. neoformans* MLST database (*12*). Environmental samples were processed and plated onto birdseed benomyl agar, incubated at 35°C, and observed for growth at 4, 7, and 14 days.

## Results

### Case-Patient Descriptions

We identified 6 cases of *C. neoformans* infection at hospital A during 2013: 4 case-patients had BSI, and 2 case-patients had respiratory specimens (obtained from BAL) that yielded *C. neoformans.* One of the patients with a BSI also had *C. neoformans* isolated from cerebrospinal fluid (CSF) and urine specimens. The positive cultures were obtained from case-patient 1 on April 6 (respiratory specimen), from case-patient 2 on April 29 (blood), from case-patient 3 on May 1 (blood), from case-patient 4 on May 9 (blood, CSF, urine), from case-patient 5 on June 12 (respiratory specimen), and from case-patient 6 on December 31 (blood).

Case-patient ages ranged from 51 to 82 years; 3 were men (Table). Underlying chronic medical conditions included diabetes in 3 patients, asthma/emphysema in 2 patients, and malignancy in 2 patients; 1 patient had metastatic lung cancer, 1 had chronic lymphocytic leukemia, 1 had chronic renal disease requiring hemodialysis, and 1 had received a kidney transplant. None were known to be infected with HIV.

**Table:** Summary of characteristics of 6 case-patients with cryptococcosis, Arkansas, USA, 2013*

Pt. no.	Age, y/ sex	Underlying condition(s)	Symptoms at initial presentation	Admission diagnosis	ICU dates, 2013	Steroid administered	*C. neoformans* culture collection date, 2013	Days from admission to *C. neoformans* diagnosis	Infection site(s)	Serum CrAg	MLST pattern	Culture date to death	Steroid treatment
1	73/F	Diabetes, metastatic non–small cell lung cancer	Cough, shortness of breath, hemoptysis for 1–2 wks	Pneumonia with respiratory failure and sepsis	Apr 6–10	No	Apr 6	1	Lung	Negative	A	5 d	None before positive culture
2	51/F	Diabetes, renal failure, hemodialysis	Shortness of breath for 1 d	Pneumonia with sepsis	Mar 15–May 1 with intermittent transfers to floor	Hydrocortisone taper	May 1	45	Blood	NO	B	3 d	Hydrocortisone for 8 d
3	78/M	Chronic lymphocytic leukemia	Weakness and failure to thrive	Severe anemia and acute renal failure	Apr 10–14	Oral prednisone	May 9	21	Blood	NO	A	2 d	Methylprednisolone for 1 d, followed by prednisone taper starting at 100 mg for 3 d, 80 mg for 3 d, 40 mg for 3 d, 20 mg for 12 d
4	67/M	Diabetes, renal transplant	Dizziness, nausea, vomiting for 2 wk	Gastroparesis soon revised to CM and BSI	Not admitted to ICU	No	May 9	1	Blood, CSF, urine	>1:512	B	~4 mo	None (not included in cohort analysis because not admitted to ICU)
5	82/M	Coronary artery disease, emphysema	Pleuritic chest pain for 1 d	Rule out myocardial infarction, soon revised to pneumonia with sepsis and respiratory failure	Jun 9–14	Hydrocortisone taper	Jun 12	4	Lung	NO	C	3 d	Hydrocortisone for 4 d
6	53/F	Well-controlled asthma	Shortness of breath and fever for 3 d	Pneumonia with respiratory failure and sepsis	Dec 9–31	Hydrocortisone and methylprednisolone	Dec 31	22	Blood	NO	C	1 d	Hydrocortisone for 8 d and methylprednisolone for 5 d
*Pt., patient; ICU, intensive care unit; *C. neoformans*, *Cryptococcus neoformans*; CrAg, cryptococcal antigen; MLST, multilocus sequence typing; NO, not obtained; CM, cryptococcal meningitis; BSI, bloodstream infection; CSF, cerebrospinal fluid.													

Case-patients were admitted with a variety of diagnoses or symptoms: 3 (50%) had pneumonia with sepsis or respiratory failure, and 1 (17%) each had severe anemia and acute renal failure; nausea, vomiting, and confusion; and chest pain. Three case-patients were directly admitted to the ICU from the emergency department, 2 case-patients were admitted to the ICU 24–48 hours after hospital admission because of new clinical deterioration, and 1 case-patient was never admitted to the ICU. Cultures yielding *C. neoformans* were obtained 1–45 days after hospital admission. In 3 of the 4 cases of *C. neoformans* BSI, case-patients had at least 1 negative blood culture before *C. neoformans* BSI was diagnosed, indicating that the disease likely developed while the patient was in the hospital. All case-patients died; 5 died within 5 days after collection of their clinical sample that yielded *C. neoformans*, 4 before culture results were available. One case-patient, a kidney transplant recipient who exhibited nausea, vomiting, and confusion, received a diagnosis of cryptococcal meningitis on the basis of a positive CSF culture and BSI and did not require admission to the ICU. He survived to hospital discharge but died several months later of unrelated causes. Two case-patients had a serum cryptococcal antigen (CrAg) test: case-patient 1, who had respiratory *C. neoformans* infection and whose CrAg test result was negative, and case-patient 4, who had meningitis and a BSI, whose CrAg test result was positive. No autopsies were performed.

### Background Rates of *Cryptococcus* spp. 

Review of microbiology laboratory records at hospital A identified a median of 2 patients (range 0–8) per year with positive *C. neoformans* cultures during 1992–2012; most were from respiratory samples. Only 4 blood cultures yielded *C. neoformans* during this time: 1 in 2004, 2 in 2007 (February and August), and 1 in 2008 (Figure). In contrast, 4 BSIs occurred during 2013, with 3 occurring within 10 days of each other. Laboratory staff and ICPs at 4 surrounding hospitals had reported no increase in the number of *C. neoformans* isolates since January 2013, indicating that the increase in cases, especially BSI cases, was only occurring at hospital A.

**Figure:**
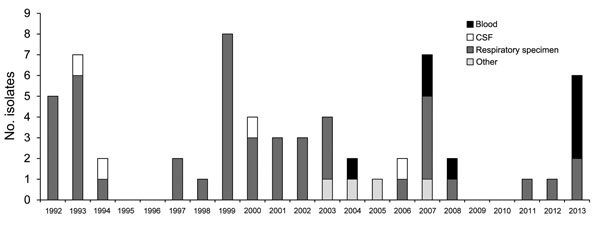
Culture-confirmed *Cryptococcus* isolates at hospital A, Arkansas, USA, 1992–2013. CSF, cerebrospinal fluid.

### Evaluation of Specimen Procurement and Processing

Detailed assessment of specimen procurement and hospital laboratory processing methods did not reveal any evidence of laboratory contamination to suggest that this was a pseudo-outbreak. Specimens from case-patients were procured by different personnel in different parts of the hospital (emergency room, medical floor) on different days. Blood cultures were processed in a closed system (BACTEC; Becton Dickinson, Franklin Lakes, NJ, USA), and in-laboratory location, growth media, and method of processing BAL and blood cultures differed.

### Evaluation for Common Exposures

Interviews with family members of case-patients did not reveal common community exposures. Case-patients lived in 4 different towns and did not work or attend religious services or social events in the same places. None of the case-patients had any recent exposure to birds, including pigeons.

Five of 6 case-patients were admitted to the ICU before their culture was positive: case-patients 1, 2, and 3 were in adjacent ICU rooms during April 6–April 14, 2013. All 5 case-patients in the ICU were also cared for by the same ICU physician; 4 of 5 had been cared for by 1 respiratory therapist (RT). No other recognized commonalities were found between case-patients in terms of procedures, devices (including hemodialysis machines and ventilators), or personnel involved in their care.

We interviewed 112 hospital staff members who might have had contact with ICU patients; 8 reported contact with birds. Types of contact included hunting pheasants, feeding backyard chickens, and maintaining outdoor bird feeders. One RT reported having 2 pet cockatiels at home with whom she spent up to 2 hours each day. The RT had cared for 4 of the 5 case-patients who were admitted to the ICU. Among the 7 other staff members with any bird contact, 2 persons, a pharmacist and an x-ray technician, had come into contact with 4 of 5 case-patients in the ICU. Sampling of hands of these 3 health care workers and a vacuum sample of the cockatiel aviary (a sunroom in the house where the birds were kept) belonging to the RT did not yield *C. neoformans.* Hospital leadership indicated that there was no concern about illegal activity or recent disciplinary action against any ICU staff members.

Interviews with facilities management staff revealed that the ICU roof had been leaking for ≈3 years in several locations, including the time case-patients 1–3 were in the ICU, before it was replaced during April 22–May 19, 2013. Notably, staff reported that the roof had been a roost for pigeons approximately 6 years earlier, and the hospital had undertaken pigeon exclusion measures at that time. Environmental swab samples taken in July 2013, ≈8 weeks after the roof replacement was completed, from the ICU rooms where case-patients 1–3 had overlapping stays did not yield *C. neoformans*. Culture of the external air filter leading into the ICU HVAC system also did not yield *C. neoformans.*

### Cohort Study to Assess Risk Factors for Cryptococcosis

A total of 1,606 patients, including 5 of 6 case-patients who had *C. neoformans *infection, were admitted to the hospital A ICU during April 1–December 31, 2013. The remaining case-patient, who had received a renal transplant and exhibited cryptococcal meningitis and cryptococcal BSI, was not admitted to the ICU and was therefore not included in this analysis.

Overall, 125 (7.8%) of the 1,606 patients admitted to the ICU during this period received some type of steroid in the ICU. Four (80%) of the 5 *C. neoformans *case-patients admitted to the ICU received steroids in the ICU before their positive culture: 2 received hydrocortisone for treatment of refractory septic shock (1 for 4 days and another for 11 days); 1 received methylprednisolone for 1 day for tumor lysis syndrome followed by an oral prednisone taper over 21 days (100 mg daily for 3 days, 80 mg daily for 3 days, 40 mg daily for 3 days, and 20 mg daily for 12 days); and 1 received methylprednisolone (5 days) and hydrocortisone (8 days) for treatment of refractory septic shock and ongoing respiratory failure. None of these patients had been receiving steroid treatment before hospital admission. Approximately 8% (121/1,601) of patients without cryptococcosis received steroids in the ICU: 7 received oral prednisone, 22 received hydrocortisone, 73 received methylprednisolone, and 19 received dexamethasone. The rate of cryptococcosis was 40.1 cases per 10,000 person-days in the ICU among persons receiving steroids, compared with 2.1 cases per 10,000 person-days in the ICU among persons not receiving steroids (rate ratio 19.1; 95% CI 2.1–171.1; p = 0.008). Exposure to the RT was also assessed as a risk factor for cryptococcosis, but no significant difference was found.

### Multilocus Sequence Typing (MLST) of Clinical Isolates

Clinical isolates from all 6 case-patients were confirmed at CDC as *C. neoformans* with 3 separate MLST patterns. Isolates from case-patients 1 and 3 had MLST patterns that were indistinguishable from each other, isolates from case-patients 2 and 4 shared a second MLST pattern, and isolates from case-patients 5 and 6 had a third MLST pattern distinct from the other 2 patterns.

## Discussion

We investigated 6 cases of cryptococcosis that occurred in 2013 in a community hospital ICU. For most of the case-patients, the disease appears to have developed while they were in the ICU after admission for other diagnoses. The patients experienced a fulminant clinical course after the diagnosis of cryptococcosis and died soon thereafter. There was no identifiable point source for the infections in the hospital or the community. Receipt of short-term steroids in the ICU was significantly associated with cryptococcosis in this cohort.

The cluster was characterized by several atypical clinical features. First, active cryptococcal disease is usually associated with HIV infection or organ transplant–associated immunosuppression. Five of 6 patients in this cluster were non–HIV-infected and nontransplant patients (NHNT); however, each did have other predisposing conditions, including chronic renal failure, chronic lung disease, hematologic malignancies, and other malignancies that might have put them at risk for cryptococcal disease (*13*). Second, *C. neoformans* BSI is extremely uncommon, especially among NHNT patients (*14*,*15*), yet 4 case-patients had blood cultures yielding *C. neoforman*s*;* only 1 case-patient (the renal transplant recipient) had meningitis, a more typical manifestation of this disease. Next, acute respiratory failure and overwhelming sepsis, as experienced by 5 patients in this cluster, are atypical manifestations of cryptococcal disease (*16*); cryptococcosis is generally a subacute infection with insidious onset of nonspecific symptoms (*5*), especially among NHNT patients, who may have prolonged symptoms before diagnosis (*17*). In contrast, the 5 NHNT patients in this cluster had a relatively short duration of symptoms and experienced respiratory failure, septic shock, or both and died within days of their positive culture.

Because *C. neoformans* infections rarely result from acute fungal exposure, and because person-to-person transmission of cryptococcosis—if it exists at all—is exceedingly uncommon (*6*,*7*), focal clusters or outbreaks of cryptococcosis are not expected and, to our knowledge, have not previously been reported. Disease usually results from reactivation of latent infection in immunosuppressed hosts; reactivation in 1 host is an independent event that is not necessarily linked to reactivation in another host. The cases we investigated were clustered in space (ICU) and time (2013, with 3 cases occurring with 10 days of each other in late April through early May). The atypical patients and the unusual clinical manifestations involved in this cluster may be an indication that the source or mechanisms of infection and disease, though not identified during the investigation, were not typical for cryptococcosis. If this were a chance clustering of independent occurrences of reactivation of latent cryptococcal infection, we would have expected to see more patients who fit the typical risk profile and have more common manifestations of the disease, and all would not have occurred at a single hospital.

We searched for a point source in both the community and hospital settings. There has been no precedent for *C. neoformans* being found in the hospital environment or being transmitted from health care worker hands, but we investigated these possibilities because they have been implicated in outbreaks with other organisms. We conducted a thorough investigation to identify any hospital sources of *Cryptococcus *spp. but did not find a hospital source through environmental assessment. However, this outcome was limited by the fact that samples were taken ≈12 weeks after the first 3 cases occurred. The meaning of the 3 different MLST patterns among the 6 case-patient isolates is unclear. This finding may be consistent either with a single point source containing multiple strains of *Cryptococcus *spp*.* (as demonstrated previously) (*17*) or a different, non–point-source cause of infection.

Although nearly two thirds of patients who seek treatment with cryptococcal disease have advanced HIV infection or have received an organ transplant (*3*), long-term oral steroid use is also a known risk factor for cryptococcal disease. In a study of cryptococcal disease among NHNT patients, the median daily dose of prednisone or prednisone-equivalent that patients were receiving was 20 mg, and the median duration of immunosuppression before cryptococcal disease was 7 months (*15*). Although use of steroids in the treatment of sepsis is generally not favored, steroids may be used for a short term in cases of septic shock not responsive to other interventions. A single 10-day tapering course of hydrocortisone used to treat refractory septic shock, as was administered to some of these case-patients, is roughly equivalent to getting 20 mg of prednisone for 15–20 days. Although cases of cryptococcal disease developing in patients receiving low-dose and short-term oral corticosteroids have been reported (*18*), clusters of cryptococcal disease in an ICU setting after receipt of short-term steroids have not been previously described. A combination of multiple chronic underlying conditions, including diabetes, renal failure, and malignancy, which are also known but less frequently associated risk factors for cryptococcosis, and short-term steroid use in the ICU may have contributed to reactivation disease or dissemination of acute infection acquired from an unknown source in the community or the hospital. Further research should be conducted to better understand the relationship between short-term steroid use and the risk for opportunistic infections, including cryptococcal disease.

This investigation has several limitations. First, we considered that all patients with positive *C. neoformans* cultures had cryptococcosis. However, the manifestations were unusual, raising the possibility that some or all case-patients might not have had true cryptococcal disease. Unfortunately, serum CrAg testing results, pathologic specimens, and autopsy findings, all of which might have helped definitively determine if true cryptococcal disease was present, were not available for most case-patients. 

Second, although we cannot definitively rule out the possibility of a pseudo-outbreak from laboratory contamination of specimens, *C. neoformans*, unlike environmental molds, is not typically a laboratory contaminant. Thus, we believed that laboratory contamination was a less likely explanation for the cluster. The findings of *C. neoformans* in different types of specimens also made laboratory contamination less likely. 

Third, we did not investigate the possibility of a common contaminated intravenous medication as a source for these infection and therefore cannot rule out this explanation as a cause of the outbreak. Again, given the different sites of infection (both respiratory and bloodstream) among case-patients, a common-source contaminated intravenous fluid also appeared unlikely. 

Finally, we were only able to look at a limited number of co-variates in the cohort study because of the reliance on an automated query of the electronic database. Individual chart review to determine severity of illness or underlying conditions was not possible for all 1,600 patients in the ICU. Therefore, we could not control for differences in reasons for admission to the ICU and severity of underlying illness.

Although we did not find a point source for infections in the hospital or community, we found that short-term steroids used in the ICU were associated with case status. To clarify whether this association between short-term steroid use and cryptococcosis is generalizable, similar studies examining rates and duration of ICU steroid use and cryptococcosis should be conducted at other hospitals. *C. neoformans* infection may need to be included in the differential diagnosis of a patient receiving short-term steroids in the ICU setting.

We recommended heightened vigilance for cryptococcal infection among ICU patients at hospital A, especially those receiving steroid treatment. We also asked that physicians carefully assess the need for steroid use in patients admitted to the ICU and weigh the risk for possible cryptococcal infection against the benefits of steroid use in each patient’s case.
